# China, or Cinchona

**Published:** 1887-05

**Authors:** J. T. Kent


### CHINA, OR CINCHONA.

### A Lecture by Professor J. T. Kent, M. D.

China is prepared from the Yellow Cinchona Bark, and diluted from the tincture. This Yellow Cinchona Bark is used also in preparation of Quinine. Cinchona is not Quinia, but contains Quinia. It has an individuality of its own that cannot be produced by any combination. The most that is known about China, or Cinchona, commonly, is its use for chills and fever. It is used in large quantities in whisky. This is the principal drug in the old-school practice for chills. China is seldom indicated in chills, taking one season with another. There are seasons when it becomes the epidemic remedy, but they are far apart. Only one season in ten here will find Peruvian Bark homœopathic to the majority of the cases of chills. We find in one season that Arnica is curing the majority of cases; in another, Eupatorium. This season China has been indicated. I have not seen a single Quinine case this season, but still the old school are using it all the same. Large doses will generally suppress the chill. This season the ruling remedy has been Eupatorium; next, Arsenicum, then Arnica, Nat. mur., and Ipecac. They have been curing chills for me since July last. I think it likely, if you would examine the clinic books of this college, where over two hundred cases have been treated, you will find that Eupatorium rules. There may have been one or two cases of China. Where the indications are followed they get well without Quinine. That simply means one thing—that the prescriptions have been correct. When the curative remedy has been given the patient recovers. It is a broad remedy outside of its chill and fever indications. A host of conditions exist which call for China, giving it a field of action in other complaints, seldom in chills. It corresponds to that condition of the body known as anæmia. This is not a symptom, but a condition—great loss of blood, protracted hemorrhage, diseases that destroy the red globules of the blood, marked by weakness, exhaustion, irritability, and nervous exhaustion, or neurasthenia (this word is of no value). From the fact that it produces this peculiar weakness of body—this peculiar anæmia—it has been found the most useful remedy for hemmorrhage; any complaint that results from hemorrhage; for "loss of vital fluids." It is a wonderful remedy for old debauchees, who become anæmic and feeble. There is a grand feature running through

China; that is the irritability that results from the pains of a peculiar character—drawing, digging, and darting. Another peculiar thing is the aggravation of these pains by touch. The slightest touch brings on these pains. Touch aggravates and brings them on if they have been quiet for some time. The increase of amount of touch increases the pains to the greatest intensity; they become almost unbearable. China has the most intense suffering; especially is this the case where great hemorrhages have occurred. Nervous exhaustion will sometimes follow opening of abscesses, seminal emissions, and uterine hemorrhages; they culminate in this irritability. Another grand feature of this remedy is that the pains, weakness, and sufferings are all aggravated in the open air and slightest draft. China patients cannot endure the slightest draft. The pains and sufferings of this remedy are made worse from touch or draft of the open air. On the other hand, they are made better by warmth and in a warm room. The head pains are made better from pressure. The patient gets headache as soon as he goes into the open air, and is relieved from warmth and in a warm room. Many remedies have headache coming on in the warm room, such as Puls., Sulph., and others. These general features run through the remedy.

There is another grand characteristic—congestion, that localizes after the loss of fluids; pneumonia, that comes on after great uterine hemorrhage; abortion or miscarriage, after a surgical operation that has been attended with much hemorrhage and violent nervous shock. China is the first remedy to be thought of under such circumstances. For localized congestion following these losses think of China always. Give it unless some other remedy is especially more appropriate. Most of the pains seem to be worse from motion. The myalgic pains seem to be, under certain circumstances, better from motion. The pains in the abdomen seem to be better from slightest motion, but this is not a very marked characteristic. Motion is not to China what it is to Bryonia and Rhus. Another prominent feature is its great flatulence. The abdomen is distended almost to bursting; abdomen is tympanitic, painful, and sore, with these tearing, cramping, darting, and digging pains; all worse from touch and slightest draft of air. A marked feature of its flatulence is that he is not in the least improved by passing of little or even considerable amount of wind by belching (Lyc.), and reverse of Carbo veg., which has relief from expulsion of flatus. China, Carbo veg., and Lycopodium stand at the head of the list for this marked flatulence, almost full to bursting. Many remedies are particularly windy, but these seem to have incarcerated flatus. In Carbo veg. the abdomen will be distended almost to bursting; when he begins to pass wind he gets relief. China gets no relief from passing flatus. Now, in China we have great debility, marked trembling of the hands, tongue, lips, and quivering and twitching of the muscles, which is called *subsultus tendinum.* You will see this state in low typhoid conditions, after marked hemorrhages, after great shock, or any exhausted condition of the body. The hemorrhages of China almost belong to the red string of the remedy, because it has produced such profuse hemorrhages. It may be large and active, yet passive and very protracted. Dark clots belong to China.

Another marked feature is the ringing in the ears. Now, this ringing appears to be very much like that which follows very marked hemorrhage (a continuous ringing in the ears after a uterine hemorrhage); face pallid, almost green. This is not a catarrhal ringing, but a nervous state, with threatened fainting (syncope). Another marked feature is its characteristic periodicity, every other day; every seventh or fourteenth day is less characteristic, but still it has it.

China has a few peculiar little symptoms that you will want to remember. They are very odd in their way; for instance, when the mother places the child to her breast she gets a toothache. You will be annoyed by that symptom if you don't know China. The mother will apply heat, which will relieve; touch will make it worse—especially is this the case in women who have had hemorrhage at their confinement. Every time she puts the child to her breast she has digging, tearing pain in a tooth or all of the teeth. (Every time the child nurses there is a discharge from the uterus: Silic.) (Every time the child nurses a sharp pain shoots to the back: Crot.-tig.) A case in this city was so bothersome and so severe as to last several weeks. Morphine, hot poultices, and many other things had been used by others without any relief. One dose of Crot. tig. cured this case. I have met this symptom about half a dozen times in the sick-room. (Cramps coming in abdomen or back every time the child nurses: Puls. and Cham.) There is one more symptom that is a peculiar one—a pain in the opposite breast while the child nurses; this is a Borax symptom. For instance, when the child is nursing the left breast, there is a violent pain in the right. These little things are peculiar and useful. There is another general symptom belonging to the general state of China, and that is a sore, bruised feeling all over the body, like Arnica, only the latter is more marked. China is indicated if this sore, bruised feeling follows a profuse hemorrhage, or any of these vital losses, or severe diarrhœa that has been profuse. (In most all other instances it would make you think of Arnica. Baptisia has it; also Actæ race., in hystero-rheumatic diatheses. Baptisia has it in relation to fevers, and here closely associated with Arnica.) Now, these symptoms thus far modify the whole text. Whether your symptoms are related to chills and fever, or one disease or another, these states modify your symptoms. It would hardly be necessary to take up the text only to show how to apply the red string. You might go over the text again and again, and not figure out this much as the ruling symptoms belonging to the text. Here it says in text, in relation to its vertigo: Vertigo after loss of animal fluids; head feels weak, can hardly hold it erect. All this belongs to this general state of anæmia of China. China is seldom indicated in the earliest conditions of acute affections, because we have other remedies more appropriate. It comes in after marked debility, destruction of the blood corpuscles, with general weakness of the body, which brings on this vertigo. The vertigo and dizziness are from great bodily weakness when China is indicated. Now, you know, there will be a throbbing headache, better from pressure and in warm room, coming on after hemorrhage, and attended with sleeplessness, after labor with flooding, after any surgical operation attended with more or less loss of blood; night after night this patient remains awake. You don't need to prescribe Morphine. Sensation as if head would burst with sleeplessness; headache worse from motion or any jar. It is not that which modifies a symptom, but that which modifies *all* symptoms which belongs to the red string. Better in the room means better from warmth and excluded from draft of air. Brain feels bruised. Many of the complaints of China are worse at night; the pains are worse at night; the diarrhœa and the sweat also. It has profuse night sweats as a strong symptom. The sweat is oily.

Face.—Habitual nosebleed, especially morning on rising. But here we find under face, heat when entering a room from the open air; pale blue around the eyes. This gives you the pallid anæmic countenance. Toothache while infant sucks the breast; toothache worse from least contact with open air or current of air; better from pressing teeth together or biting hard. Another grand feature, that the exhaustive sweats are accompanied by thirst. China has thirst during the sweat of its peculiar fever. In typhoid, when the tongue becomes black and cracked and bleeding, you will very often need China for that state. (Arsenic has very dark, brown tongue, sometimes black. Arnica and Baptisia have very dark, black tongue.) Another marked feature under desires and aversions: longing for dainties (cake and pie); aversion to fat things and warm food; to warm food and drinks, which disagree with the stomach. Aggravations from warm things, like Puls. and Phos. (Puls. is immediately made sick from warm tea and food. Phos. vomits food and drink as soon as it gets warm in the stomach; the pregnant woman gets sick and vomits from placing her hands in warm water: Phos.) The vomit of this remedy is sour and worse at night. Blackish, bloody vomiting is very much like China. Crotalus and Lachesis have produced black vomit; also Arsenicum. China has a vomit looking like inky water. This class of remedies, China, Arsenicum, Arnica, Crotalus-hor., and Lachesis will most frequently be your choice for black vomit in yellow fevor (also Hydrocyanic acid.)

Cold feeling in the stomach is a peculiar symptom. These symptoms are all associated with the exhaustive sweats, as perhaps second in a characteristic way. Constant satiated feeling; yet can eat, but feels worse afterward; this occurs in the anæmic state, after great losses and sinking of strength. (Lycopodium has a keynote; after he has taken a mere mouthful he feels as full as if he had eaten a whole meal.) China has this with exhausted feeling, or after he had had chills and fever for some time. Fullness in the stomach and bowels; belching does not relieve. (Lyc. and Carbo veg.) Another prominent feature is slow digestion; food remains long in the stomach, especially if eaten too late in the day. (Puls., the food remains long in the stomach and sours; spits it up.) China is worse in the open air and Puls. is better; this decides the choice.

There is only one state, so far as I know, where the patient is made better by a draft of air, and that is in its collapse, when he wants to be fanned; he is covered with the sweat, often exhausting hemorrhages, and threatened collapse; China, Carbo veg., Ars., Secale. Vomiting of blood, great loss of blood; stomach sensitive to touch; gastralgia after depletion; acid belching of food; relief from motion; stomach feels sore, as if ulcerated; cannot bear slightest touch. China also produces enlargement of the liver and spleen. If you have a general anæmic state and history of Quinine drugging, you must antidote effects of the Quinine, and then give China high. Gastro-duodenal catarrh, after loss of fluids; colic from gall-stones; swollen, hard liver; these symptoms must be tied to the general characteristics of the remedy. The diarrhœic stool is generally painless and at night.

Cinchona will serve you very universally in diarrhœas that are profuse and watery—yellow watery, or yellow, thin, and fecal. Black, watery diarrhœa especially calls for China, Arsenicum, Secale, and Baptisia. It is characteristic of China to have a painless involuntary stool of a cadaverous odor. The diarrhœa, in a characteristic way, is generally worse in the night and after meals, and a peculiar symptom in relation to it is at times a profuse sweat and then a violent thirst. You will find in Bell's little work on *Diarrhœa* that China is laid down for thirstless diarrhœa; but it has thirst if there is marked sweat; and, again, quite characteristic, thirst while at stool. (Arsenicum has a characteristic thirst before and during stool; also a marked thirst when the desire to go to stool comes on, attended with dryness of the mouth and thirst.) China has this same desire for water while sitting at stool. (Capsicum has thirst immediately after stool.) Another marked feature in relation to the painless nightly diarrhœa is lienteria. It is a common occurrence in infants to pass curdled milk in the stool. Another grand feature belonging to the aggravation of the diarrhœa: it is worse after a meal or after eating; sometimes worse in the mornings in hot weather and from fruits and sour beer; and that which belongs to China in general, from exhaustive diseases and discharges. Sometimes before and sometimes during stool, thirst; some colic, but it is the exception. When it is present, it is most likely to occur before stool. It is somewhat like Colocynth, relieved by doubling up. Another prominent feature is that the stool is commonly frothy. If you have a patient taken down any time during the night with a violent, gushing diarrhœa, stool being black or dark brown, cadaverous smelling, and attended with great exhaustion and pallor, stool driving out of bed frequently during the night, marked thirst during the sweating period (the more sweat the more thirst), painless stool, and may contain undigested lumps, very exhausting, give China. In the morning, forenoon, and during the day he may be somewhat better. Stool rather scanty during the day—a chronic diarrhœa that has a nightly driving out of bed, worse in the hot weather, after eating, and at night. A China diarrhœa, chronic, painless, and attended with lienteric stools, associated with the fevers and febrile states, with congestion of the liver, and stomach disorders, and with jaundice, will have these urinary symptoms, turbid, dark, or white sediment, and becoming cloudy, it deposits a yellow sediment, scanty greenish sediment. Another peculiar

14

feature, where they have taken whisky in large quantities, there will be a sediment of almost a mahogany color; it lies in rings around the bottom of the vessel. (For mahogany deposits always think of Phytolacca; a clay-colored deposit that requires soap stone to get it off will call for Sepia; it may occur in intermittents, but more commonly is associated with uterine disorders. Sepia has a gray, shining deposit, leaving a water line.) In an inflammation of the ovaries that follows hemorrhage or abortion, you will want to know China; the parts are very sensitive to touch; there may be tumid abdomen and pains worse at night; the patient generally is worse from the slightest draft of air. You must remember to apply this exhaustive state that comes from hemorrhages. China will often cure a dropsy that is the result of hemorrhage. An old woman died in the "Memorial Home" recently who had been given up to die two years ago. When I was elected as physician of the Home I found two old ladies given up to die. One of them, in particular, had vomited an enormous amount of blood (the Matron said "two gallons"); it produced considerable exhaustion. She sent for the doctor, who treated them in vain. Dropsy came on; the limbs became œdematous; the abdomen was full of serum and very tumid. Every day, for four days, she got one dose of China, and no more medicine. In four weeks the dropsy was all gone. She was upward of eighty years when I prescribed for her. Doctors say that old people cannot get well of dropsy. She had no more medicine until a few weeks ago, when inflammation of the liver set in, her abdomen filled up, and the other day she died. The old lady lived these three years entirely free from dropsy.

Of course, we don't find in China a dropsy in a pathognomonic way, but it produces a state like unto that following a hemorrhage. No matter how the anæmic state comes on, its symptoms are, nevertheless, similar to those which China is capable of producing. Dropsy in women after abortion, with profuse hemorrhage, convulsions after uterine hemorrhage, don't need any other medicine than China high. Metrorrhagia; blood dark; fainting; convulsions; congestion of the uterus; fullness, pressing, heaviness, worse from walking. This congestion of the uterus is most likely to come on as a result of hemorrhage or some exhausting disease if China is indicated. Cocculus had leucorrhea instead of menses, but think of China also when these peculiar conditions are present.

There is a grand characteristic of China in labor—the pains cease from the hemorrhage. In this case she cannot have the hands touched (aggravation from touch). Thirst sometimes comes on immediately after the hemorrhage, like the convulsions. Another peculiar indication in the last symptom, asphyxia of the new-born after violent hemorrhage of the mother. The mother has been having a series of hemorrhages, such as are found in placenta prævia, and the child is born asphyxiated. Then give both the mother and the child China. Some chest symptoms are marked, as great rattling in the chest, threatened paralysis of the lungs of old people, another good symptom in relation to breathing; China has cold breath. (You may place Carbo veg. first, Veratrum second, and China third.) The breath is cold in collapse; for instance, Carbo veg. has a peculiar picture—the patient cannot lie down in asthmatic state, or in collapse of cholera, or from hemorrhage (China), it may be; this threatened cold stage of the body, wherein it is related to shocks, the patient is bolstered up with pillows and covered with profuse sweat and pale; the breath is cold and patient must be fanned all the time. In Veratrum there is terrible exhaustion; cold, profuse sweat; great beads of cold sweat rolling out all over the body. Now, China is a sort of cross between these medicines. If this condition has been brought on after exhaustive flooding, surgical accident, or patient is in a profoundly anæmic state, it has cold breath, exhaustion, and sweat of the other two remedies. Arsenicum ought to be mentioned in relation to this collapse. It has terrible exhaustion, but in cholera morbus, where the countenance is cadaverous and covered with sweat, it has great restlessness and exhaustion. The restlessness is peculiar in Ars.; collapsed, he has passed through the period wherein the restlessness had been present; he feels that if he should move a finger he would die, so terribly exhausted is he. Ars. is not aggravated from touch like China. It seems in the most protracted state of China there are digging and tearing pains from touch, not found in Verat., Carbo veg., and Arsenicum. There is a peculiar cough in connection with China, dry cough in the night (here again you can see the aggravation at night), with granular expectoration during the day. Cough is made worse by exposure to a draft of air. The China patient wants to be covered up warmly, even around the neck. Any little draft aggravates the cough. Rumex has a dry cough at night when lying down, and worse from the cool atmosphere of the room; he even covers the head up; coughs worse on going into the open air (also Phos.). Many remedies appear to have a cough at night, while the aggravation is little different from the mere fact of being in the night. China will have cough whether up or lying down. (Drosera has a cough as soon as the head touches the pillow.) Puls. has a cough on lying down; as soon as the head touches the pillow sometimes a cough comes on; also cough aggravated from warmth of bed. (Manganic acid has cough relieved by lying down.)

In dry, spasmodic, or suffocative night cough, as from vapor of sulphur, with bilious vomit, the patient coughs and coughs until it seems that he has worn out the mucous lining of the throat, and then comes the bloody mucous expectoration. Coughs worse with the head lying low (Arsenicum, Bry., Puls., and a host of remedies have this symptom); least draft of air; loss of fluids also after being awakened is sometimes characteristic; as soon as he rouses up from sleep he commences to cough. The chest is so sensitive that he cannot bear the least percussion. There are shooting, digging, and stitching pains in the chest. In a general way China is often restless, wanting to change the position. One hand is icy cold and the other warm (compare Ipec. and Digit.). (Lyc. has one foot cold and the other warm. This is not in organic disease, but simply a nervous state.) There are many pains, some of them attended with swelling in the limbs, but the marked feature is aggravation from handling or touch. The darting, tearing pains in the limbs are aggravated from touch, or worse from contact, as the text has it. Now and then pains in all the limbs, but especially in lower limbs, which depart in a warm room. Most generally with the pain in the limbs there is great restlessness, with some improvement on motion (not the marked improvement of Rhus); especially is this the case when the pains are not attended by swelling. Study the nervous symptoms closely. Under the nervous symptoms we have a recapitulation of the rest of the symptoms. There are a few peculiar things in relation to chill, fever, and sweat. The most marked feature before the chill is the thirst, the next is great restlessness the night before the chill. Unlike many remedies, there is no characteristic time of the chill. The most marked time of periodicity is every other day—it is marked in this periodicity. The next most characteristic feature is thirst during the profuse sweat. Few remedies have this. It is more common for the China chill to occur in the afternoon or in the evening or night. Another marked feature is heat of the face while there is yet coldness of the body, with dilated veins (remember that Cham. and Arnica have hot face and cold body). China, in a characteristic way, seldom has thirst during the chill, seldom thirst during the heat, but marked thirst during the sweat. It has thirst for little water and often, like Arsen., but also for large quantities often. A peculiar feature is that in the fever he wants to throw the covers off, but as soon as he does he becomes chilly. (Nux cannot bear to be uncovered in any stage; the least lifting of the covers makes him chilly.) The special feature is exhausting sweat with thirst. In relation to chill, he wants to be near a stove, but it increases the chill.

